# Comparing the prehospital NEWS with in-hospital ESI in predicting 30-day severe outcomes in emergency patients

**DOI:** 10.1186/s12873-022-00598-5

**Published:** 2022-03-14

**Authors:** Peyman Saberian, Atefeh Abdollahi, Parisa Hasani-Sharamin, Maryam Modaber, Ehsan Karimialavijeh

**Affiliations:** 1 Prehospital and Hospital Emergency Research Center, Tehran University of Medical Sciences, Tehran, Iran; 2 Anesthesiology Department, Imam Khomeini Hospital Complex, Tehran University of Medical Sciences, Tehran, Iran; 3 Tehran Emergency Medical Service Center, Tehran, Iran; 4 Department of Emergency Medicine, Sina Hospital, Tehran University of Medical Sciences, Tehran, Iran

**Keywords:** Emergency medical services, Early warning score, Triage, Emergency severity index, Patient outcome, Smartphone

## Abstract

**Background:**

In Iran, the emergency departments (EDs) have largely adopted the emergency severity index (ESI) to prioritize the emergency patients, however emergency medical services (EMS) mainly triage the patients based on the paramedics’ gestalt. The National Early Warning Score (NEWS) is a recommended prehospital triage in the UK. We aimed to compare prehospital NEWS and ED ESI for predicting severe outcomes in emergency patients.

**Methods:**

An observational study was conducted in a university-affiliated ED between January and April 2021. Adult patients who arrived in the ED by EMS were included. EMS providers calculated the patients' NEWS upon arriving on the scene using an Android NEWS application. In the ED, triage nurses utilized the ESI algorithm to prioritize patients with higher clinical risk. Then, Research nurses recorded patients' 30-day severe outcomes (death or ICU admission). Finally, The prognostic properties of ESI and NEWS were evaluated.

**Results:**

One thousand forty-eight cases were included in the final analysis, of which 29 (2.7%) patients experienced severe outcomes. The difference between the prehospital NEWS and ED ESI in predicting severe outcomes was not statistically significant (AUC = 0.825, 95% CI: 0.74–0.91 and 0.897, 95% CI, 0.83–0.95, for prehospital NEWS and ESI, respectively).

**Conclusion:**

Our findings indicated that prehospital NEWS compares favorably with ED ESI in predicting 30-day severe outcomes in emergency patients.

## Background

Triage is an essential part of emergency medical care both in the field and in the emergency department (ED) [1]. In emergency patients, it is shown that severe outcomes, including death, intensive care unit (ICU) admission, organ damage, disabilities, and other morbidities, are closely related to delays in providing proper emergency medical care for the patients.[2] These adverse outcomes will profoundly affect the cost of treatment, length of hospital stay, and put pressure on the whole health care system [3].

In the prehospital setting emergency medical services (EMS) providers are faced with a wide-range of restrictions including time constraints, environmental hazards, lacking diagnostic facilities, and work-related psychological pressure. Therefore, utilizing a valid scale for identifying high-risk patients in the prehospital setting is important [4].

In 1997, Morgan et al. introduced an Early Warning Score (EWS) for identifying critical patients [1]. Ten years later, the National Early Warning Score (NEWS) was described by the Royal College of Physicians in the United Kingdom (UK) [5]. It consists of six quantitative measures of the patients' physiologic parameters, including systolic blood pressure, heart rate, respiratory rate, temperature, peripheral oxygen saturation, and level of consciousness. A higher NEWS score, indicates a higher-risk in the patients [6]. Additionally, the application of NEWS is convenient and requires a short training [7]. In 2017, NEWS2 was introduced to focus on patients with hypercapnic respiratory failure and is currently recommended for in-hospital and prehospital triage in the UK [8].

To date, many studies have evaluated the prognostic value of NEWS in the ED [9–14]. Nevertheless, prehospital NEWS still requires further research. A recent review has shown little published evidence regarding the effectiveness of utilizing NEWS by ambulance services [15].

In Iran, all EDs use the emergency severity index (ESI) to direct emergency patients in different areas of the ED, including resuscitation room, observation unit, fast track, and treatment areas. ESI originates from the United States (US) and consists of five triage levels based on the patients' presentation, vital signs, and the number of resources they might need in ED [16]. ESI score ranges between 1 and 5 and lower score indicates a higher urgency [17–19].

ESI Guidelines recommend regular education and quality control to maintain and improve the reliability of the ESI [20]. Accordingly, Studies in the EDs that implemented a regular ESI education and audit, have shown a better interrater reliability of ESI scores (weighted Kappa between 0.75 and 0.89) [18, 21]. In contrast, in studies that did not mention these factors, the interrater reliability of ESI is lower [20, 21].

In Iran, EMS providers are mainly anesthesiology and operating room technicians. These technicians have completed a two-year accredited program in colleges. Also, recently, medical universities have started a two-year program specifically for training EMS providers. In comparison, Nurses undergo a different education and training through participating in a four- year accredited education. So, in Iran, the sphere of education varies between the nurses and the EMS providers. Considering these differences, ESI triage would not be a suitable triage tool for the Iranian EMS providers.

When it comes to providing the optimized care possible for emergency patients, relying on the clinical gestalt alone would be a subject for debate because all clinicians are prone to bias in decision-making, especially in emergencies [22].

In this study we sought to compare the ED ESI with the prehospital NEWS in detecting emergency patients who are more likely to experience death or ICU admission.

## Methods

### Design and setting

Between January and April 2021, an observational study was conducted at the Tehran EMS center and a university-affiliated urban ED in Tehran, Iran. The ED has an average annual census of 60,000 patients. Our ED does not usually receive children (age under 16) because in Iran; the designated children's hospitals are responsible for evaluating the children. Also, our hospital is a level-1 trauma center, and traumatic cases are more frequent in our ED. Among EMS stations in Tehran, four EMS stations that were linked to our ED were selected.

All emergency patients who were transferred to the ED were assessed and followed to find out their 30-day outcome. Sampling was performed during the weekdays from 9 am to 6 pm. Four research nurses were recruited to collect the data and follow up with the patients. They recorded the patients' demographic data (age, gender, traumatic or non-traumatic cause of the event), ESI level, and NEWS. An emergency medicine physician trained research nurses how to gather and record data in a checklist prepared by researchers. The local ethics committee approved the conduct of the study. All data was recorded anonymously and with respect to patients' privacy. Also, informed consent was obtained from the patients, relatives, or legal guardians upon the ED admission for follow up evaluations.

### Participants

All adult patients (age ≥ 16 years) brought to the ED by EMS providers were included. We excluded the patients who left the ED against medical advice, transferred to other medical centers, had missing triage data or lost follow-up, and were confirmed dead at the scene or upon ED arrival.

### Data collection

Since utilizing NEWS2 requires knowledge regarding the blood gases, which is beyond the scope of our paramedics, we opted to evaluate the NEWS in our prehospital patients [23].

EMS providers calculated the Patients' NEWS based on their initial observations in the field. They used a downloadable NEWS app installed on their smartphones to calculate the NEWS (https://play.google.com/store/apps/details?id=com.gumptionmultimedia.newsscore). Since this app was not linked to any database, they recorded patients' NEWS in a checklist prepared by the researchers and handed the checklist to the research nurses in the ED.

Upon the ED arrival, triage nurses reevaluated the patients using the ESI algorithm to prioritize patients with higher clinical risk. Finally, research nurses recorded patients' ESI levels into the checklists.

Before embarking on the study, all EMS providers underwent a 2-h group training session held at the Tehran EMS center. They all installed the downloadable NEWS application on their smartphones and used it during the workshop to calculate the NEWS in simulated cases. The workshop was supervised by two board-certified emergency medicine physicians who were familiar with the different triage scales.

ED triage nurses evaluated the ESI level upon the patient's arrival to the ED.

In many EDs, the EMS-reported assessments and vital signs is an integral role of ED triage. In Iran, however, ED nurses rely more on their primary assessments and reevaluate all the patients regardless of the EMS providers' reports. In our ED, Triage nurses undergo regular ESI education and training. Also, their work is monitored by senior ED nurses to ensure a high-quality triage.

Thirty days after the ED arrival, research nurses reviewed the patients' medical records to identify the severe outcomes. They also followed up with the patients who were discharged from the hospital by phone call to confirm 30-day severe outcomes related to the index event.

### Variables

The NEWS parameters were blood pressure (mm Hg), pulse rate ( per minute), respiratory rate (per minute), body temperature (°C), Oxygen saturation (SPO_2_), and level of alertness measured by AVPU (Alert, Verbal, Pain, Unresponsive).

Based on the final NEWS score, patients' clinical risk was determined as NEWS 0–4: low risk, NEWS 5–6: medium risk, and NEWS ≥ 7: high risk [24]. In addition, patients with a high score in a single parameter (Score over 3 in any NEWS elements) were considered as low-moderate risk [25–28].

ESI algorithm is a five-level triage that categorizes the patients based on the provider's assessments. ESI levels are level 1 (Patients who require immediate life-saving intervention), Level 2 (high-risk situations, confused, lethargic, disoriented, severe pain or distress), level 3 (patients who need more than one ED resource), level 4 (patients who need one ED resource), Level 5 (patients who need zero ED resource). In addition, before allocating a patient to ESI level 3, the nurse checks the patient's vital signs (SPO_2_, PR, RR), and If the vital signs are abnormal, the triage nurse may upgrade the triage to ESI level 2 [19].

### Data measurement

EMS providers used analog sphygmomanometers, portable pulse oximeters, and non-contact infrared digital thermometers to measure the NEWS variables. After recording these variables, they entered them into their smartphone NEWS application to calculate the final NEWS.

In the ED, nurses used their gestalt and ESI algorithm to determine the ESI levels. Also, they used a digital cardiac monitor installed in the tirage room to measure vital signs for ESI level 3 patients. The final ESI levels were recorded in the patients' triage form. Research nurses recorded these scores into their checklists. Triage nurses were blinded to the patients' NEWS scores.

### Outcome

The main outcome was the agreement between the prehospital NEWS and the ED ESI in detecting patients who were more likely to experience severe outcomes (NEWS ≥ 7 and ESI levels 1 and 2).

### Sample size

There was no similar study for comparing prehospital NEWS with ED ESI. With a presumed interclass correlation coefficient of 20% between prehospital NEWS and ED ESI in 95% of confidence interval 1418 patients were required to participate in the study. Also, the sample size required for accuracy testing, based on the assuming of 85% sensitivity for each tool, 2% of severe outcomes among transported patients in our ED, an error of 15% to estimate the sensitivity, and a type 1 error of 5%, the minimum required sample size was 1088. Therefore, based-on previous data and statistics of ED patients we estimated that three months of the ED patient flow was sufficient and near to the required sample size.

### Data analysis

Chi-square test and Fisher test were used to compare the severe outcomes between the patients with high and medium NEWS or ESI with the patients who had low risk news or ESI. Also, to assess the amount of agreement between ESI by ED nurses and NEWS by EMS technicians, we redistributed ESI scores from five to three tiers as high risk ( ESI level 1,2), moderate risk (ESI level 3), and low risk (ESI level 4,5). Pearson correlation was used to assess the strength of the relationship between the ESI and NEWS. The prognostic properties of ESI and NEWS in terms of 30-day severe outcomes were evaluated using the receiver operating characteristic (ROC) curve. The 95% confidence interval for AUC-ROC calculated based-on the DeLong method and the test equality of AUC-ROC assessed based-on Chi-square test.. P-value < 0.05 indicated statistical significance. Analyses were performed using Stata version 15 ( StataCorp. 2017. Stata Statistical Software: Release 15. College Station, TX: StataCorp LLC).

## Results

**Fig. 1:**
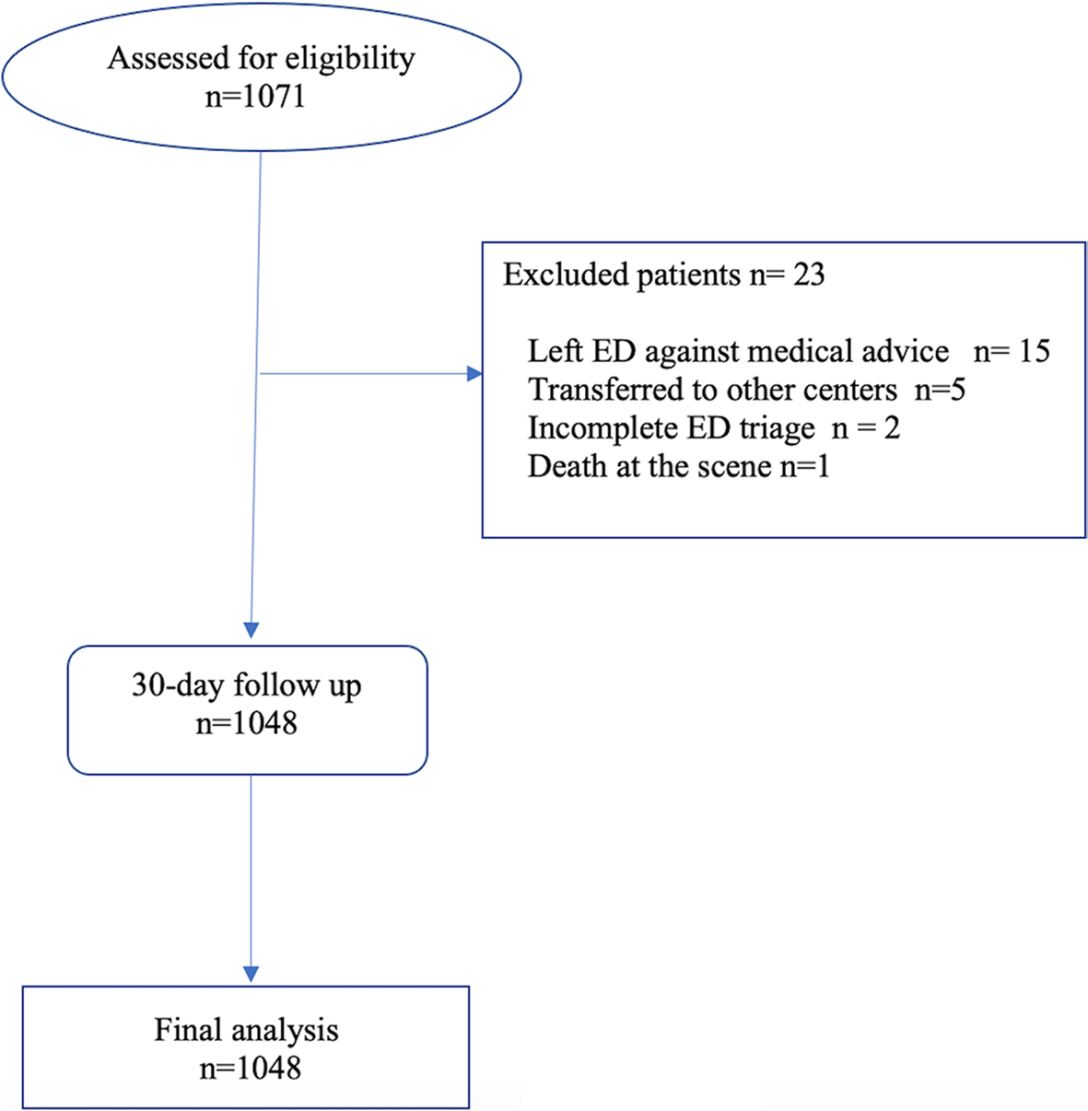
Flow chart of the study

A total of 1914 patients were transferred to the target ED by four EMS stations. 1071 patients were assessed by research nurses, of which 643(60.03%) were male, and 428 (39.96%) were female. The mean ± SD age of participants was 45.1 ± 19.7 (Table 1). During the study, 23 (2.1%) patients were excluded (11 patients left the ED against medical advice, four patients were transferred to other medical centers, two patients had missing triage data, one patient was declared dead on the scene, and five patients were excluded during the follow up because they did not respond to phone calls) (Fig. 1). Among the excluded patients, 18 cases were low risk, three patients were medium risk, and one was high risk based on the NEWS.

**Table 1:** Characteristics of the study population

Variable	Value
Age (Year, Mean ± SD)	
Gender n (%)	45.1 ± 19.7
Male	643 (60.03)
Female	428 (39.96)
Prehospital vital signs (Mean ± SD)	
Systolic Blood pressure (mmHg)	120.4 ± 20.7
Diastolic Blood pressure (mmHg)	76.2 ± 10.3
Pulse rate (per minute)	83.5 ± 25.2
Respiratory rate (per minute)	16.5 ± 1.9
Oxygen saturation (%)	95.6 ± 2.2
Body temperature (C°)	36.8 ± 0.8
Level of consciousness n(%)	
Alert	1035 (96.65)
Verbal	15 (1.4)
Pain	10 (0.93)
No response	11 (1.02)
Chief complaint n(%)	
Trauma	590 (55.08)
Non-Trauma	481 (44.91)
30-day Outcome, n (%)	
ED discharge	653 (62.3)
Ward admission	366 (34.9)
ICU admission	14 (1.33)
Death	15 (1.46)

Seventy-one (6.77%) patients had NEWS ≥ 7, and the mean ± SD NEWS was 2.72 ± 2.38 (Table 2).

**Table 2:** Participants' prehospital NEWS

NEWS	Clinical risk	Number (%)
0–4	Low	880 (83.96)
Red score*	Low Medium	21 (2.0)
5–6	Medium	76 (7.25)
≥ 7	High	71 (6.77)

^*^ 3 in a single parameter

One thousand forty-eight patients were included in the final analysis, of which 29 (2.7%) cases experienced severe outcomes (death or ICU admissions). Among patients with severe outcomes, 25 (86%) cases had medical illnesses, and 4 (13.7%) patients had traumatic injuries.

In patients with medium and high risk NEWS, 143 (13.6) participants experienced adverse outcomes, while in low risk NEWS 4 (0.38) patients had severe outcomes (P value = 0.001).

In other words, patients with medium and high NEWS had a higher frequency of death or ICU admission [25 (31.7) for medium and high risk NEWS vs 4 (0.45) for low risk NEWS] (Table 3).

**Table 3:** Frequency of severe outcomes in patients with low, medium, and high NEWS

Outcome n (%)	NEWS (Clinical risk)			NEWS score (Mean ± SD)
	**0–4 (Low) *n* = 880**	5–6 (Medium) /Red score* *n* = 97	** ≥ 7 (High) *n* = 71**	
ICU admission	2 (0.23)	4 (4.1)	8 (11.26)	5.07 ± 2.09
Death	2 (0.22)	5 (5.1)	8 (11.26)	7.08 ± 4.61
Death and ICU admission	4 (0.45)	9 (9.27)	16 (22.5)	6.86 ± 4.4

^*^ 3 in a single parameter

In the ED, ESI triage was performed in 1048 patients, of which 789 (75.3%) patients were identified as ESI level 3.

In patients with medium and high risk ESI, 29 (2.7) participants experienced severe outcomes, while in low risk ESI none of the patients had sever outcomes (P value = 0.001).

Patients with ESI levels 4 and 5 (low-risk patients) had a lower frequency of severe outcomes (Table 4).

**Table 4:** Frequency of severe outcomes in patients based on the ESI levels

Severe outcome n (%)	ESI Level			
	**1** ***n* = 13**	**2** ***n* = 179**	**3** ***n* = 789**	**4 and 5** ***n* = 67**
ICU admission	2 (15.4)	11(6.1)	1 (0.1)	0 (0)
Death	8 (61.53)	5 (2.79)	1 (0.12)	0 (0)
Death and ICU admission	10 (76.9)	16 (8.93)	3 (0.38)	0 (0)

The Pearson correlation coefficient between NESWS and ESI was 0.25, indicating a weak linear correlation between the two triage tools.

**Fig. 2:**
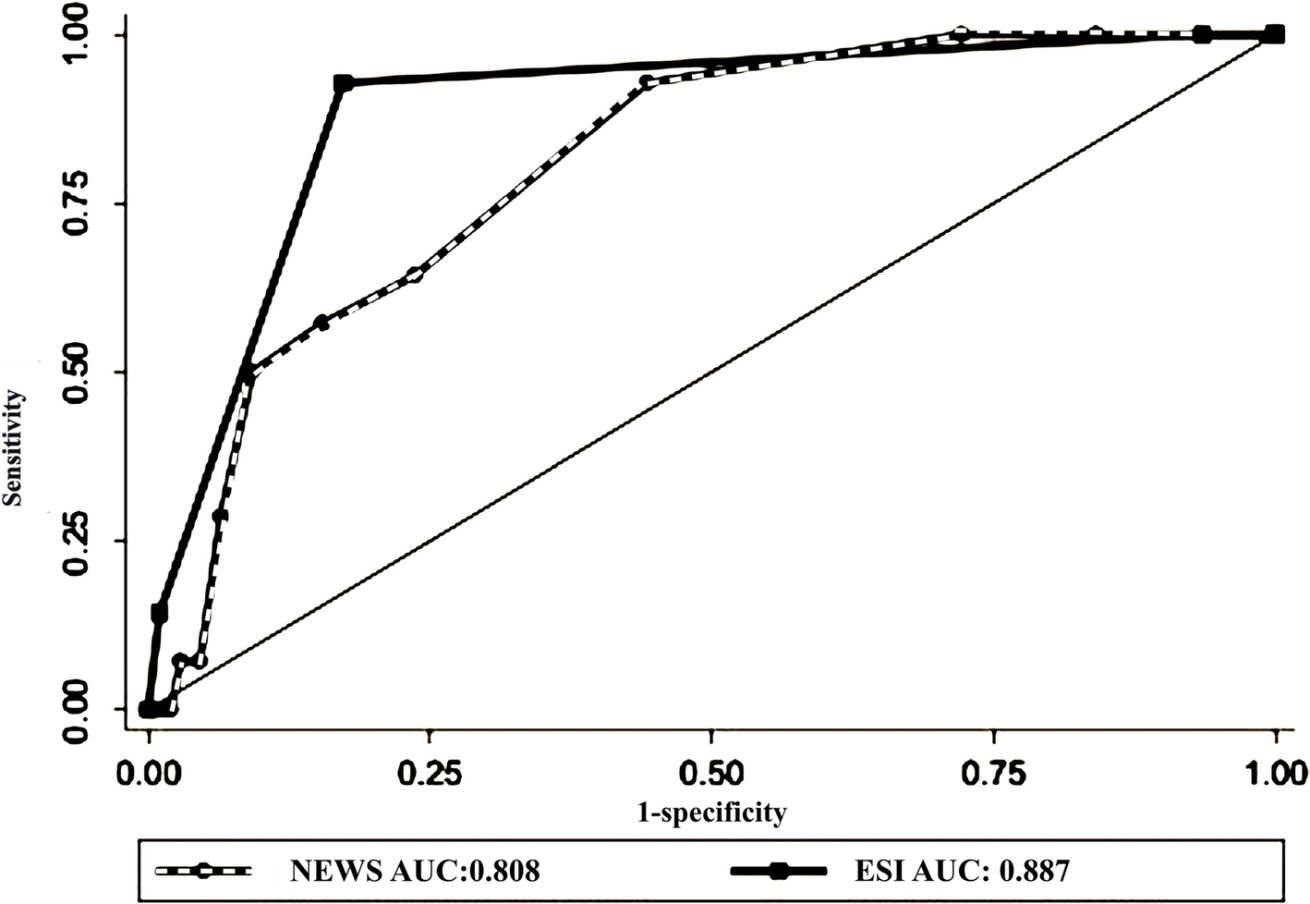
The receiver operating characteristic curve for predicting ICU admission by Prehospital NEWS and ED ESI

**Fig. 3:**
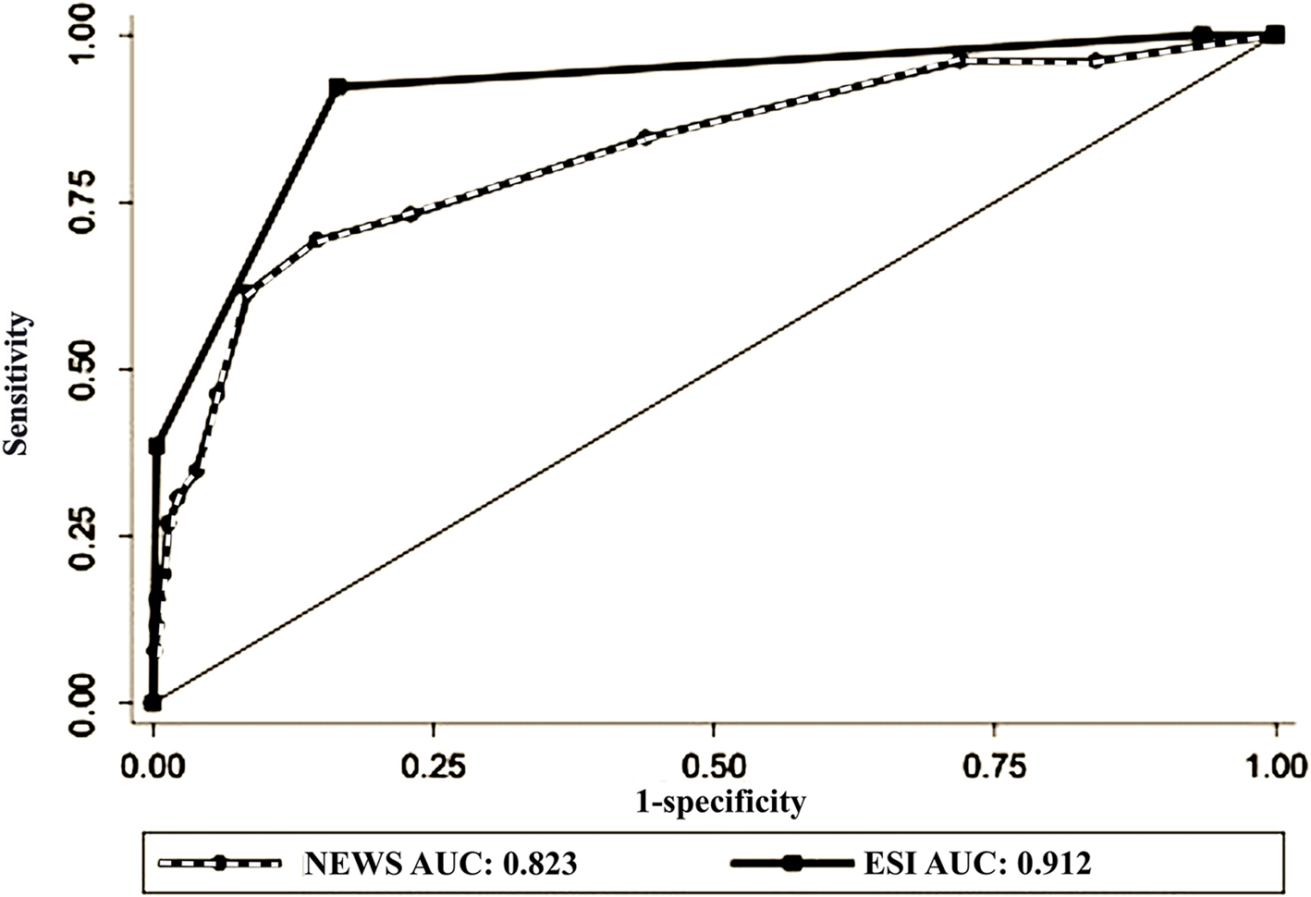
The receiver operating characteristic curve for predicting in-hospital mortality by Prehospital NEWS and ED ESI

In predicting ICU admission, the area under a receiver operating characteristic curve (AUC) for NEWS and ESI was 0.8 ( 95%CI: 0.71–0.89) and 0.88 (95%CI: 0.81–0.95), respectively (P-value = 0.11),(Fig. 2). For mortality, the AUC of NEWS and ESI was 0.82 (95% CI: 0.82–0.91) and 0.91 (95%CI:0.85–0.96), respectively (Fig. 3).

**Fig. 4:**
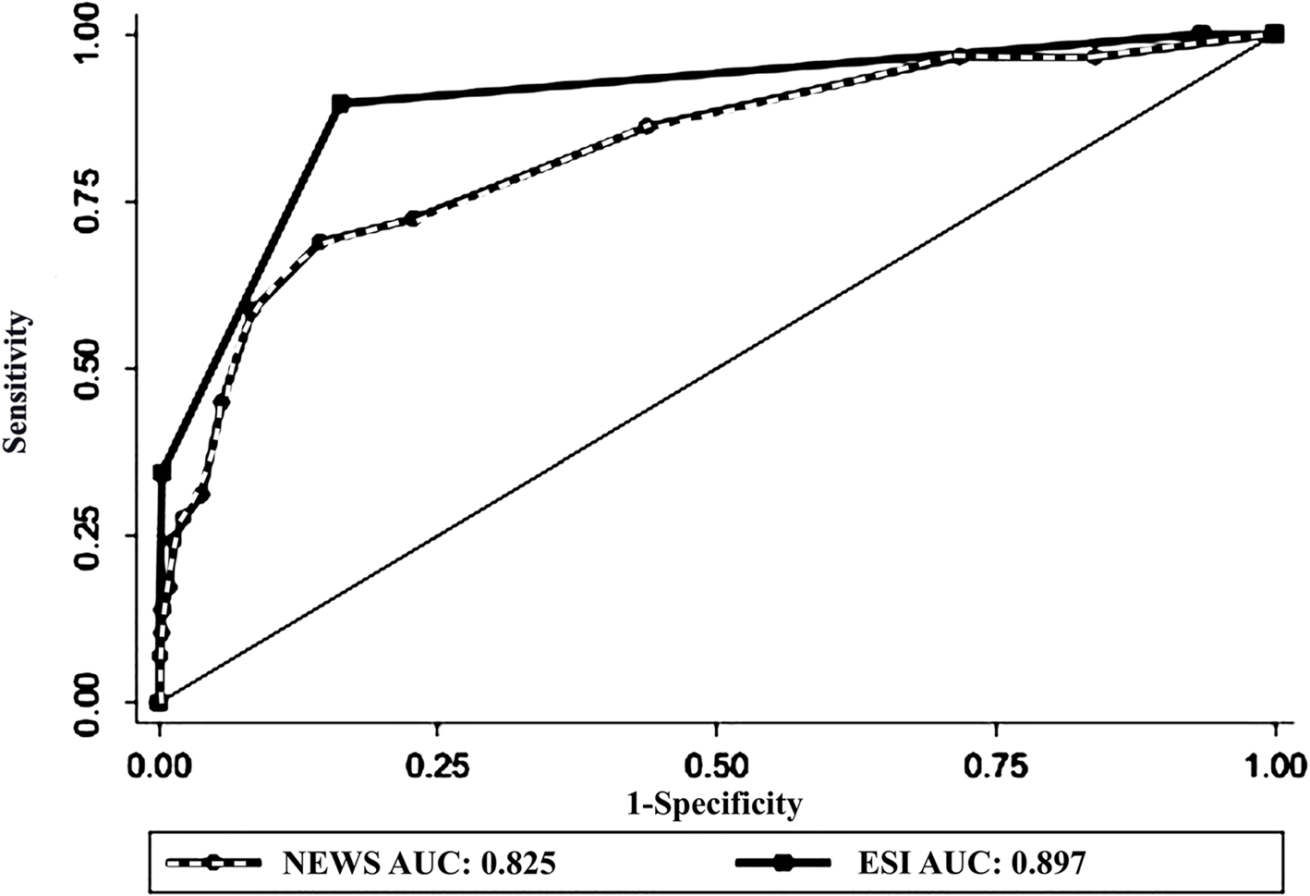
The receiver operating characteristic curve for predicting severe outcomes (death and ICU admission) by Prehospital NEWS and ED ESI

For all outcomes (death and ICU admission), the difference between the two measures in predicting severe outcomes was not statistically significant (AUC = 0.825, 95% CI: 0.74–0.91 and AUC = 0.897, 95% CI, 0.83–0.95, for prehospital NEWS and ED ESI, respectively)(Fig. 4, and Table 5).

**Table 5:** sensitivity, specificity and predictive value of NEWS and ESI for sever outcomes in the participants

% (95% Confidence interval)	NEWS	ESI
Sensitivity	86.2 (68.3–96.1)	89.6 (72.6–97.8)
Specificity	85.97 (83.7–88.04)	83.4 (81–85.6)
Positive predictive value	11.1 (9.2–13.4)	9.7 (8.4–11.7)
Negative predictive value	99.67 (99.1–99.8)	99.75(81.2–85.7)

## Discussion

In Iran, similar to the US, the ESI triage is well-established in the EDs [20]. Therefore, ED staff and EMS providers are not familiar with the NEWS triage; Also, in Iran, EMS does not utilize prehospital triage tools to identify high-risk patients [29]. In this study we sought to determine the accuracy of prehospital NEWS as a standard prehospital triage tool. First, 1048 patients underwent prehospital triage by EMS providers using the NEWS. Then, the clinical risks identified by the prehospital NEWS were compared with ED ESI levels. Also, patients were followed up over 30 days to determine severe outcomes including death or ICU admission. During the follow-up, 29 patients experienced severe outcomes. Data analysis revealed a significant association between the higher prehospital NEWS and severe outcomes.

Several studies have shown that NEWS can help EMS providers in identifying high-risk patients [30, 31]. For instance, one study in Finland with 12,426 cases and another study in Japan with 2847 patients assessed the association between prehospital NEWS and patients outcomes in the ED [32]. Both of them reported that prehospital NEWS could reliably identify high-risk patients who are more likely to suffer from life-threatening conditions [32, 33].

Our results indicated that In predicting ICU admission, AUC for NEWS and ESI were 0.8 and 0.88, respectively. Also, for 30-day mortality, the AUC of NEWS and ESI was 0.82 and 0.91, respectively. For all severe outcomes (death + ICU admission), the AUC of prehospital NEWS and ED ESI was not significantly different (0.82 and 0.89 for prehospital NEWS and ESI, respectively). These results are similar to prior studies that reported the same predictive properties of NEWS for ICU admission (AUC ranged between 0.67 and 0.85) and death ( AUC of 0.67 to 0.84) in patients with sepsis, pneumonia, respiratory distress, and exacerbation of chronic obstructive pulmonary diseases [10, 12, 34–37].

Furthermore, in the present study, ED ESI levels 1–3 were associated with severe outcomes. Finally, prehospital NEWS and ED ESI were comparable in predicting the 30-day severe outcomes in emergency patients. There were no similar studies to compare this finding, but our results are in accordance with other studies that have compared ESI with in-hospital NEWS. Phungon et al. reported that ESI and in-hospital NEWS were comparable in predicting ICU admission or death in septic patients. They reported that ESI (levels 1 and 2) was 96.7% sensitive in predicting in-hospital mortality in patients with sepsis [38].

Although the agreement between the two triage tools in determining the clinical risks of the patients was weak in our study, some factors might explain this difference. Our EMS technicians calculated prehospital NEWS immediately when they arrived on the scene and before any other medical intervention. So, transport time and medical interventions by EMS technicians (oxygen therapy, pain management, and intravenous fluid infusion, to name a few) have probably affected patients' physiologic parameters in the ED. A study conducted by Abbot et al. compared prehospital NEWS with ED admission NEWS and found a moderate correlation between these time points. They reported that ambulance NEWS was higher than ED NEWS in most cases, indicating improved patients' hemodynamics upon ED arrival [36].

In addition, our research showed that paramedics could easily calculate the NEWS using a NEWS android application installed on their smartphones. Considering the brief education we provided for our paramedics and the fact that calculating the NEWS does not require additional skills, it can be argued that paramedics may benefit from utilizing the NEWS triage in their missions [15]. In addition, the primary beneficiary of utilizing a standardized prehospital triage tool are the patients who may receive appropriate care in a timely manner.

NEWS may also help in assigning a proper medical team for different emergency situations such as requesting an advanced response team, summon more experienced paramedics, or decreasing the transfer time as much as possible. Also, it may help to select the best receiving hospital which can meet the patients' needs and level of urgency. additionally, it could help in anticipating the involvement of the critical-care staff and preparing ICU beds upon patients arrival at the receiving hospitals [39].

### Limitations

This study has several limitations. First, prehospital NEWS was calculated based on the patients' initial physiologic status. Upon ED arrival, patients' hemodynamic parameters were influenced by EMS medical interventions (oxygen therapy, pain control, immobilization, and intravenous fluid infusion) and transfer time. Therefore, ED nurses were likely to obtain different hemodynamic data from the patients.

Second, we conducted this study in a single ED, making our study vulnerable to selection bias. Also, patients were included between 9 am and 6 pm; this is likely demonstrated by a young skew to the age of our patients (mean of 45 years old). So it should be considered as another source of bias in this study.

Additionally, our hospital is a referral trauma center and this has impacted the male to female ratio in our study.

Last but not least, while the current guidelines recommend NEWS2 as an ideal prehospital triage, due to the limited equipment in our ambulances (lack of blood gas analyzers), we did not use NEWS2 in this study.

## Conclusions

Our findings indicated that prehospital NEWS compares favorably with ED ESI in predicting 30-day severe outcomes in emergency patients. Future research is needed to evaluate the effect of utilizing prehospital NEWS on patient care.

## Data Availability

The data that support the findings of this study are available on request from the corresponding author.

## Abbreviations

EDs: Emergency departments
EMS: Emergency medical services
NEWS: National Early Warning Score
ICU: Intensive care unit
ESI: Emergency severity index
SPO_2_: Oxygen saturation
ROC: Receiver operating characteristic
